# Efficient conversion of phytosterols into 4-androstene-3,17-dione and its C1,2-dehydrogenized and 9α-hydroxylated derivatives by engineered *Mycobacteria*

**DOI:** 10.1186/s12934-021-01653-9

**Published:** 2021-08-16

**Authors:** Xin Li, Tian Chen, Fei Peng, Shikui Song, Jingpeng Yu, Douanla Njimeli Sidoine, Xiyao Cheng, Yongqi Huang, Yijun He, Zhengding Su

**Affiliations:** 1 Key Laboratory of Industrial Fermentation (Ministry of Education), National “111” Center for Cellular Regulation and Molecular Pharmaceutics and Hubei Key Laboratory of Industrial Microbiology, Hubei University of Technology, Wuhan, 430068 China; 2 Hubei Goto Biotech Inc., No. 1 Baiguoshu Road, Shuidu Industrial Park, Danjiangkou, 442700 Hubei China

**Keywords:** 1,4-Androstadiene-3,17-dione (ADD), 21-Hydroxy-20-methylpregn-4-en-3-one (BA), 3-Ketosteroid-1,2-dehydrogenase (KstD), 3-Ketosteroid-9α-hydroxylase (Ksh), 4-Androstene-3,17-dione (4-AD), 9α-Hydroxyl-4-androstene-3,17-dione (9OH-AD), *Mycobacterium *sp. strain

## Abstract

4-Androstene-3,17-dione (4-AD), 1,4-androstadiene-3,17-dione (ADD) and 9α-hydroxyl-4-androstene-3,17-dione (9OH-AD), which are important starting compounds for the synthesis of steroidal medicines, can be biosynthetically transformed from phytosterols by *Mycobacterium* strains. Genomic and metabolic analyses have revealed that currently available 4-AD-producing strains maintain the ability to convert 4-AD to ADD and 9OH-AD via 3-ketosteroid-1,2-dehydrogenase (KstD) and 3-ketosteroid-9α-hydroxylase (Ksh), not only lowering the production yield of 4-AD but also hampering its purification refinement. Additionally, these 4-AD industrial strains are excellent model strains to construct ADD- and 9OH-AD-producing strains. We recently found that *Mycobacterium neoaurum* HGMS2, a 4-AD-producing strain, harbored fewer *kstd* and *ksh* genes through whole-genomic and enzymatic analyses, compared with other strains (Wang et al. in Microbial Cell Fact 19:187, 2020). In this study, we attempted to construct an efficient 4-AD-producing strain by knocking out the *kstd* and *ksh* genes from the *M. neoaurum* HGMS2 strain. Next, we used *kstd*- and *ksh*-default HGMS2 mutants as templates to construct ADD- and 9OH-AD-producing strains by knocking in active *kstd* and *ksh* genes, respectively. We found that after knocking out its endogenous *kstd* and *ksh* genes, one of these knockout mutants, HGMS2^*Δkstd211 + ΔkshB122*^, showed a 20% increase in the rate of phytosterol to 4-AD conversion, compared relative to the wild-type strain and an increase in 4-AD yield to 38.3 g/L in pilot-scale fermentation. Furthermore, we obtained the ADD- and 9OH-AD-producing strains, HGMS2^*kstd2 + Δkstd211+ΔkshB122*^ and HGMS2^*kshA51 + Δkstd211+ΔkshA226*^, by knocking in heterogenous active *kstd* and *ksh* genes to selected HGMS2 mutants, respectively. During pilot-scale fermentation, the conversion rates of the ADD- and 9OH-AD-producing mutants transforming phytosterol were 42.5 and 40.3%, respectively, and their yields reached 34.2 and 37.3 g/L, respectively. Overall, our study provides efficient strains for the production of 4-AD, ADD and 9OH-AD for the pharmaceutical industry and provides insights into the metabolic engineering of the HGMS2 strain to produce other important steroidal compounds.

**Supplementary Information:**

The online version contains supplementary material available at 10.1186/s12934-021-01653-9.

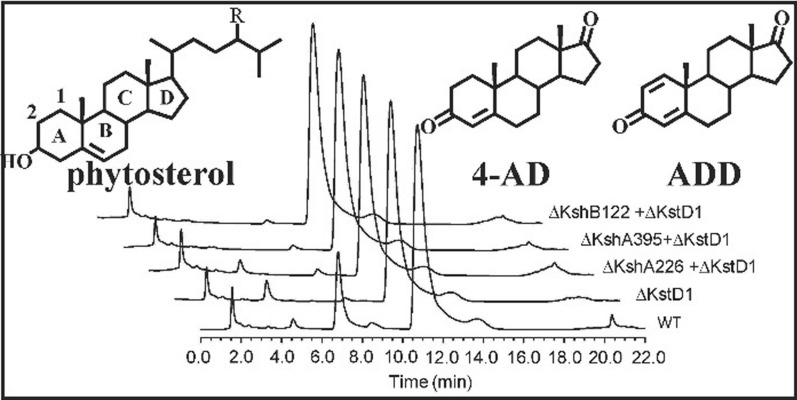

## Introduction

Steroids are widely used as antibacterial, anti-inflammatory, antiviral and anticancer therapeutics [1, 2]. The core structure of steroids consists of three six-member cyclohexane rings and one five-member cyclopentane ring. 4-Androstene-3,17-dione (4-AD), 1,4-androstadiene-3,17-dione (ADD) and 9α-hydroxyandrost-4-ene-3,17-dione (9OH-AD) are the most important starting materials for the synthesis of advanced steroid compounds. Accordingly, these three compounds are in high demanded by the pharmaceutical industry [3, 4]. The bioconversion of phytosterol to 4-AD, which is an important process in the pharmaceutical industry, uses bacteria that harbor partial steroid-degrading pathways [5, 6]. Although many microbes such as *Pseudomonas *sp. NCIB 10590 [7] and *Nocardioides simplex* VKM Ac-2033D [2, 8] contain steroid-degrading pathways, only strains from the *Mycobacterium* genus can efficiently accumulate 4-AD for potential industrial application [1, 3, 9, 10]. *Mycobacterium neoaurum* NRRL B-3805 was the first strain that was used to industrially transform phytosterol to 4-AD via its incomplete steroid-degrading pathways [11, 12]. Since then, other mycobacterial strains with the potential industrial application in the biotransformation of phytosterol to 4-AD, have been identified, including *Mycobacterium* sp. NwIB-01 [10]. Nevertheless, the accumulation of 4-AD in currently available industrial 4-AD-producing strains is often accompanied by the occurrence of ADD, 9OH-AD and other 4-AD derivatives generated during phytosterol transformation [1, 4]. The presence of these 4-AD derivates not only hampers the 4-AD purification and refining process but also significantly reduces 4-AD yield. However, no effective ADD- and 9OH-AD-producing strains are currently available for industrial-scale application.

Biochemical and genomic investigations have revealed that all steroid-degrading bacteria share a core pathway via 9,10-secosteroid intermediates that participate in the breakdown of steroid ring structures [13, 14]. The conversion of phytosterol to 4-AD involves 11 enzymes in 14 consecutive enzymatic steps that must occurs before 4-AD can be completely degraded [15, 16]. Therefore, 4-AD can be considered a catabolic intermediate in the phytosterol-degrading pathway and a precursor of 9,10-secosteroid intermediate. Two enzymes, 3-ketosteroid-1,2-dehydrogenase (KstD) and 3-ketosteroid 9α-hydroxylase (Ksh), are responsible for the rupture of the 4-AD ring at the C9,10-position. This leads to the formation of 3-hydroxy-9,10-secoandrost-1,3,5(10)-triene-9,17-dione (HSA), which is readily catabolized to CO_2_ and H_2_O [17–19].

Reducing KstD and Ksh activities in 4-AD-producing strains has been shown to prevent the ring opening of steroidal intermediates and improve the accumulation of 4-AD, ADD and 9OH-AD during phytosterol transformation [20–32]. Traditional mutation approaches including ultra-violet light and chemical-mediated mutation have been employed to improve the productivity of 4-AD and other sterol compounds [33–39]. Owning to progress in the genome sequencing of sterol degradation strains, recent studies have focused on engineering highly efficient phytosterol-transforming strains using gene and metabolic engineering. Knockout of the *casC* gene from *Rhodococcus jostii* RHA1 resulted in the accumulation of a 5-carbon side chain cholate metabolite [40]. Additionally, depletion of the *choD*, *hsdD* and *kstD* genes in *M. smegmatis vaccae* enhanced the accumulation of 9OH-AD [33]. Knockout of the *kstd* gene from the *M. neoaurum* NwIB-01 strain increased AD and ADD production [10], while knockout of the *kstd* genes from *M. neoaurum* ATCC25795 harboring highly active Ksh enzymes led to the accumulation of 9OH-AD [23, 30]. However, to the best of our knowledge, these strains have not been reported for industrial application because of the existence of multiple and even unknown KstD and Ksh enzymes in these strains.

KstD is a flavin adenine dinucleotide (FAD)-dependent enzyme that introduces a double bond at the position between the C1 and C2 atoms of the A-ring of 3-ketosteroid substrates including 4-AD and 9OH-AD [41]. A bioinformatic study revealed that KstD enzymes exist in more than 500 different microbes, especially in bacteria belonging to the phylum Actinobacteria [41]. Moreover, a single species may contain multiple KstDs that are substrate specific. For instance, *Rhizobium sp.* strain [42], *M. neoaurum* DSM 1381 [43, 44] and *Gordonia neofelifaecis* NRRL B-59395 [45] carry at least three different KstD enzymes, each of which exhibits unique features in terms of its amino acid sequence, three-dimensional conformational structure and substrate specificity. Ksh is a two-component iron-sulfur monooxygenase that consists of the oxygenase KshA and the reductase KshB. The Ksh homologues are distributed over several phylogenetic groups [46]. For example, *Mycobacterium *sp. VKM Ac-1817D effectively transforms phytosterol into 9OH-AD using five KshAs and two KshBs [47]. Additionally, *R. rhodochrous* DSM43269 contains five KshA homologues, each of which exhibits distinct specificity for different substrates [48–50]. Many putative *ksh* genes have been identified in currently available 4-AD-producing strains [10, 51]. The balance between the accumulation of 4-AD and the inhibition of 4-AD degradation during phytosterol fermentation is managed by controlling the fermentation time. However, it is difficult to control this balance during prolonged fermentation processes, which often results in unstable conversion yield [16, 52]. This drawback becomes even worse when phytosterol fermentation is conducted in an aqueous medium [53, 54]. Accordingly, it is essential that an ideal 4-AD producing strain lacks all KstD and Ksh activities for industrial application.

**Fig. 1:**
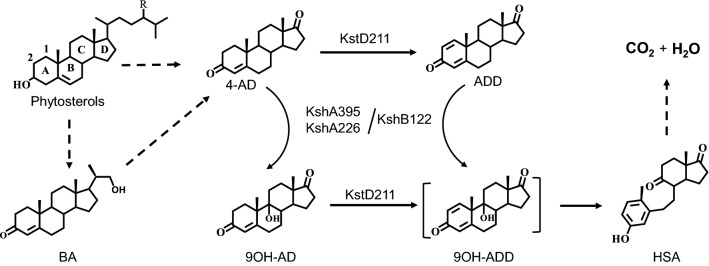
KstD and Ksh enzymes are responsible for 4-AD accumulation and its degradation during phytosterol catabolism in *M. neoaurum* HGMS2. The steroidal core structure contains four rings, namely, rings A, B, C, and D, labeled on phytosterol. Successive steps involve the formation of 9α-hydroxy-1,4-androstadiene-3,17-dione (9OH-ADD), which is a transient intermediate and simultaneously converts to 3-hydroxy-9,10-secoandrost-1,3,5(10)-triene-9,17-dione (HSA). 4-AD: 4-androstene-3,17-dione; ADD: 1,4-androstadiene-3,17-dione; 9OH-AD: 9α-hydroxy-4-androstene-3,17-dione and BA: 21-hydroxy-20-methylpregn-4-en-3-one

We recently characterized a 4-AD producing strain, *Mycobacterium neoaurum* HGMS2, using genomic analysis and found it contained less *kstd* and *ksh* genes than other investigated strains. Specifically, the genome of the HGMS2 strain contains one *kstd* gene (*kstd211*), two *kshA* genes (*kshA226* and *kshA395*) and one *kshB* gene (*kshB122*) [55]. The enzymes encoded by these genes, KstD211, KshA226, KshA395 and KshB122, are relevant to the generation of 4-ADD and 9OH-AD and the degradation of 4-AD during phytosterol fermentation (Fig. 1). In this study, we investigated the effects of these four genes on the accumulation of 4-AD during phytosterol transformation, using a homologous recombination approach, with the goal of providing efficient 4-AD-, ADD- and 9OH-AD-producing strains for industrial application. Knockout of the *kstd211* and *kshB122* genes from the HGMS2 strain abolished the accumulation of ADD and 9OH-AD and significantly improved the conversion yield of 4-AD compared with that of the wild-type strain. Furthermore, we knocked in heterogenous active *kstd* or *ksh* genes to selected HGMS2 mutants to generate ADD- and 9OH-AD-producing strains. As a result, our study work provided a high-efficiency 4-AD-producing bacterial strain and also provides ADD- and 9OH-AD-producing strains with potential application in the pharmaceutical industry.

## Materials and methods

### Materials

A DNA gel extraction kit was purchased from Omega Biotek (Hubei, China). Other molecular biology reagents were of the highest grade and were obtained from Thermo Scientific (Shanghai, China). 4-Androstene-3,17-dione (4-AD), 1,4-androstene-3,17-dione (ADD), 9α-hydroxyl-4-androstene-3,17-dione (9OH-AD), 21-hydroxy-20-methylpregn-4-en-3-one (BA) and phytosterol were obtained from Hubei Goto Pharmaceutical Co. (Xiangyang, China). In this study, the purity of phytosterol obtained from commercial source was 95% on average and typically, composed of β-sitosterol (47.5%, MW = 414.71 Da), campesterol (26.4%, MW = 402.7 Da), stigmasterol (17.7%, MW = 412.69 Da) and brassicasterol (3.6%, MW = 398.66 Da) and so that an average of molecular weight of 410.40 Da. Phenazine methosulfate (PMS, 99%) and nitro blue tetrazolium with purities of 99% were purchased from Sigma-Aldrich (Shanghai, China). Restriction enzymes, dNTPs, and *Taq* polymerase were purchased from TaKaRa Co. (Dalian, China).

### Bacterial strains, plasmids, DNA extraction and purification

*Mycobacterium neoaurum* HGMS2, a non-pathogenic industrial *Mycobacterium*, was maintained in our laboratory and deposited at China Center for Type Culture Collection (CCTCC No: M2012522) [55] and its genome sequence was deposited in GenBank (CP031414.1). The plasmids p2NIL (Cat. 20188) [56] and pT18mobsacB (Cat. 72648) were purchased from AddGene (Watertown, MA, USA).

The genomic DNAs of *Mycobacterium *sp. HGMS2 and its mutants were extracted using a Bacterial Genomic DNA Extraction Kit from Tiangen (Beijing, China). Plasmids were purified using a Plasmid Purification Kit from Tiangen (Beijing, China). PCR fragments were purified using an agarose gel purification kit (Qiagen, USA).

### Construction of homogenous recombinant vectors and knockout and knockin experiment

The pT18mobsacB was used as a template to amplify the sucrose lethality gene *SacB* that was originally from *Bacillus subtilis* 168. *SacB* and its promotor and terminator were amplified by PCR using two primers (sacB-F and sacB-R) that introduced *Eco*RI sites at both ends. The nucleotide sequences of sacB-F and sacB-R were 5′-CCGGAATTCCACATATACCTGCCGTTC-3′ and 5′-CCGGAATTCTTATTTGTTAACTGTTAATTGT-3′, respectively. The PCR fragment and the p2NIL plasmid were individually digested with the *Eco*RI restriction enzyme. The SacB fragment and the restriction plasmid were recovered by agarose gel purification. Digested *SacB* DNA fragment was subcloned into the digested p2NIL plasmid by T4 DNA ligase, and the resultant plasmid was named p2NILSac.

To knock out the *kstd* and *ksh* genes from the HGMS2 genome, each of these genes was replaced through homologous recombination with a specific DNA fragment that contained the upstream sequence and the downstream sequence outside of the targeted gene. Each upstream sequence and each downstream sequence were approximately 1 kbp in length. The upstream sequence and the downstream sequence of each target gene were amplified from the HGMS2 genome (Additional file 1: Table S1) using two pairs of primers (Additional file 1: Table S2). The amplified upstream sequence and downstream sequence for each target gene were digested with two pairs of restriction enzymes, *Bam*HI/*Xba*I and *Xba*I/*Hin*dIII, respectively. Digested fragments were ligated into the p2NILSac vector that was predigested with *Bam*H I/*Hin*dIII. The ligated plasmids were transformed into *E. coli* DH5a competent cells for amplification. Constructed plasmids were confirmed by DNA sequencing.

The same strategy was used to knock in active *kstd* and *ksh* genes. The *kstd2* gene from *Mycobacterium neoaurum* DSM 1381 (Additional file 1: Figure S1) [44] was amplified by PCR using the primers KstD-OF and KstD-OR (Additional file 1: Table S3). The amplified *kstd2* fragment and the plasmid p2NIL-SacB with *kstd* upstream and downstream homology arms were treated with *Xba*I and digested products were purified with PCR clean-up kits. The digested *kstd2* gene fragment and plasmid were ligated using T4 DNA ligase to obtain the *KstD2* knockin vector. The ligation mixture was transformed into *E. coli* DH5a for amplification and DNA sequencing. The *kshA* knockin vector was constructed in the same manner as the above except for its template. A *kshA* gene was adopted from *kshA51* (Additional file 1: Figure S2) [57] and chemically synthesized. The synthetic *kshA51* gene was ligated with the plasmid p2NIL-SacB with *kshA226* upstream and downstream homology arms to obtain the *kshA*51 knockin vector.

### Screening of recombinant mutants

DNA electroporation was performed with a Gene Pulser Xcell System (BioRad, Hercules, CA, USA) according to a protocol described by *Goude* et al. [58]. The competent cells were prepared by the following procedure. The bacteria were cultured in 50 mL of LB medium containing 0.05% Tween-80 until the OD_600nm_ value reached 0.4. The cells were collected by centrifugation and washed three times with 10% glycerol containing 0.05% Tween-80. Cell pellets were then resuspended in 100 µL of 10% glycerol solution and used as competent cells. Then, 5 ng of recombinant plasmid was added to 100 µL of competent cells, and the mixture was transferred to a 0.2 cm precooled electroporation cuvette. A single pulse of 2.5 kV, 25 µF and a resistance of 1000 Ω with a constant wave pulse was usually used. The time constant was set for 23 ms. The bacterial suspension was diluted to 1 mL with LB medium and incubated for 3 h at 30 °C. The culture was then spread on an LB agar plate containing 50 mg/ml kanamycin (LBK) and allowed to grow at 30 °C for 3 days. Colonies were screened by PCR for positive recombination.

A few colonies growing on LBK plates were picked, and each colony was inoculated into 5 mL of LBT medium (0.05  Tween-80 in LB) containing 50 mg/mL of kanamycin and cultured at 30 °C and 200 rpm for 2 days. The cell culture was diluted in 10^3^- and 10^5^-fold with LBT medium. Then, 100 µL of diluted cell suspension was spread on LBK agar plates containing 10% sucrose (LBKS), and 100 µL of diluted cell suspension was also spread on LBK agar plates as a control. Both types of plates were incubated at 30 °C for 3 days. When the number of colonies on the LBKS plates was less than that on the LBK plates, colonies were picked from the LBKS plates for PCR verification. Positive colonies were transferred to 5 mL of LBT medium and cultured at 30 °C and 200 rpm for 2 days. Their genomic DNAs were extracted for PCR verification. The verified mutant strains were stored at − 80 °C.

### Small-scale fermentation

The wild-type and mutant *Mycobacterium* strains were initially cultured in 5 mL of LB medium at 30 °C for two or three days till its OD_600nm_ value reached 13–15. The culture is used as seeds and was inoculated into a 100 mL of fermentation medium that contains yeast extract (8 g/L), glycerol (6 g/L), (NH_4_)_2_HPO_4_ (0.6 g/L), NaNO_3_ (2 g/L), phytosterol (10 g/L), F-35 (20 g/L), β-cyclodextrin (5 g/L), and lectin (3 g/L), and shaken at 30 °C and 200 rpm. Then, 1 ml of fermentation broth was collected every 24 h for 7 days for the extraction of metabolites.

The fermentation broth was thoroughly mixed with ethyl acetate at a ratio of 1:1. The mixture was centrifuged at 8000×*g* for 5 min and the supernatant was collected. An aliquot of the supernatant was directly used for thin layer chromatographic (TLC) assay. The supernatant was collected and dried by heating using a hair drier. The dried sample was dissolved in 40% methanol solution for high-performance liquid chromatographic (HPLC) assays.

### Molecular cloning, expression and purification of KstD2 and KshA51 enzymes

Preparation of KstD211 and KshA395 were described previously by Wang et al. [55]. The synthetic KstD2 and KshA51 genes were subcloned through *Bam*H I and *Eco*R I into a modified pET28b vector that its thrombin cleavage site was replaced with a Tobacco Etch Virus (TEV) protease cleavage site. Each plasmid was individually transformed into *E. coli* BL21(DE3) cells for protein expression. Protein expression and purification of KstD2 and KshA51 enzymes were performed according to previously-reported procedure [55]. Purified KstD2 and KshA51 enzymes were assayed with SDS-PAGE as a single band and used for activity assay.

### 3-Ketosteroid-1,2-dehydrogenase activity assay

The activity of 3-ketosteroid-1,2-dehydrogenase (KstD) was measured spectrophotometrically at 600 nm for 10 min at 30 °C using 5-Methylphenazinium methosulfate (PMS) and 2,6-Dichlorophenolindphenol (DCPIP) as artificial electron acceptors. The reaction was conducted in 500 µL of mixture solution containing 50 mM Tris-HCl buffer (pH 7.0), 1.5 mM PMS, 40 µM DCPIP and 250 mM AD in 2% methanol. The reaction was initiated by adding 20 µg of purified KstD211 or KstD2. A reaction lacking AD was monitored as a control. Activity is expressed as U/mg of protein after subtracting control data. One unit (U) of enzyme activity is defined as the amount of enzyme required for reduction of 1 µmol of DCPIP per minute (ε_600 nm_ = 18.7 × 10^3^ L/mol·cm).

### 3-Ketosteroid-9a-hydroxylase activity assay

The assay of 3-ketosteroid-9a-hydroxylase (Ksh) activity was performed by a method modified from a procedure published by Petrusma et al. [50]. Briefly, the reaction mixture contained 500 µL of air-saturated 50 mM Tris-HCl buffer (pH 7.0), 100 µM NADH and 20 µg of enzyme. The reaction was initiated by the addition of 250 µM 4-AD into the mixture. The substrate solution was prepared by dissolving 4-AD powder in methanol. All assays were performed at 25 °C. A kinetic program on a NanoDrop C2000 spectrophotometer was used to continuously record NADH oxidation at 340 nm (ε = 6.22 L/mol cm).

The enzymatic conversion of 4-AD to 9OH-AD was also evaluated by monitoring substrate and product concentrations using an HPLC system equipped with a Waters 2945 UV detector at 215 nm and a 150 × 4.6 mm C18 reverse-phase 5 μm ODS-analytical column. The mobile phase contained 40% methanol and 60% water and the flow rate was set at 1 ml/min. In this work, the elution times for 4-AD and 9OH-AD were 10.6 and 4.4 min, respectively. The concentrations of 4-AD and 9OH-AD were calculated from their respective peak areas using standard curves of 4-AD and 9OH-AD, respectively. The *R*^*2*^ value for the standard curve was 0.99.

### TLC and HPLC analyses of fermentation metabolites

TLC assays were performed on 0.25-mm-thick silica gel that impregnated with fluorescent dye (Haiyang Chemical Co., Qingdao, China). Sample extracts were spotted in 5 µL aliquots onto silica TLC plates. The TLC plates were developed with a solvent system containing petroleum ether and ethyl acetate at a ratio of 6:4 (v/v) as the mobile phase. The products of the enzymatic reaction on TLC plates were visualized under ultraviolet (UV) light. If needed, the TLC plates were stained with 20% sulfuric acid at 100 °C for 10 min to identify compounds that were invisible under UV light.

HPLC analysis was carried out on a C_18_ reverse-phase Sunfire column (5 μm, 4.6 × 150 mm, Waters, USA) at 30 °C with a Waters HPLC system equipped with a UV detector. Extracted metabolites dissolved in ethyl acetate were transferred to a clean glass vial and dried in vacuo. Dried powder was redissolved in methanol (approximately 1 mg/mL), and the solution was then filtered through a 0.22 μm microporous membrane. HPLC was performed using a mixture of methanol and water at a ratio of 60:40 (v/v) as the mobile phase at a flow rate of 1 mL/min. Analytes were detected with UV light at 214 nm. The identities of HPLC peaks were confirmed by comparison with the standards of 4-AD, ADD, and 9OH-AD. Each peak area was integrated with software provided by Waters and used to evaluate the compound concentration.

The conversion rate (*Conv*) of phytosterol to 4-AD, ADD and 9OH-AD was estimated using the following equation:

$$Conv=\frac{\frac{Mst}{MWst}}{\left(\frac{Mp}{MWp}\right)} \%,$$

where *M*_*st*_ and *Mp* are the weights of steroid and phytosterol, respectively. *MW*_*st*_ and *MWp* are the molecular weights of steroid and phytosterol, respectively. In this work, the average molecular weight of phytosterol is 410.40 Da (see above).

### Mass spectrometric analysis of fermentation metabolites

LC/MS mass spectra of HPLC fractions were obtained under isocratic or gradient elution conditions, with acetonitrile:water (1:1) as the mobile phase. Small percentages of formic acid were added to the water mobile phase in some experiments to check the effects of the additives on the sensitivities and the mass spectra of the steroid compounds. After choosing the best conditions, i.e., acetonitrile:water = 60:40 with 0.2% formic acid, the detection limit was assessed for each fraction using selected ion monitoring of the most abundant ions in the spectra.

### Pilot-scale fermentation

Pilot-scale fermentation was conducted in a 15 L fermenter. The fermentation medium consisted of yeast extract (8 g/L), glycerol (6 g/L), (NH_4_)_2_HPO_4_ (0.6 g/L), NaNO_3_ (2 g/L), phytosterol (80 g/L), F-35 (20 g/L), β-cyclodextrin (20 g/L) and lectin (3 g/L). Phytosterol was mixed with glycerol and β-cyclodextrin, and thoroughly emulsified before the mixture was transferred to the fermenter. All of the other materials were added into the fermenter, and water was added until the total volume of the medium reached 10 L. The medium was autoclaved in situ at 121 °C for 30 min, followed by cooling down to 30 °C with stirring at 500 rpm. The fermentation medium was inoculated with 1% (v/v) of the seed that was cultured for 2 days. The fermentation was processed at 30 °C with stirring at 500 rpm and the dissolved O_2_ concentration (50–60%) and the pH value (pH 7.5) of the fermentation broth were constantly monitored in-line and automatically adjusted if necessary. Samples were collected every 6 h to monitor the concentration of 4-AD, ADD and 9OH-AD.

## Results

### Characterization of HGMS2 fermentation extracts

**Fig. 2:**
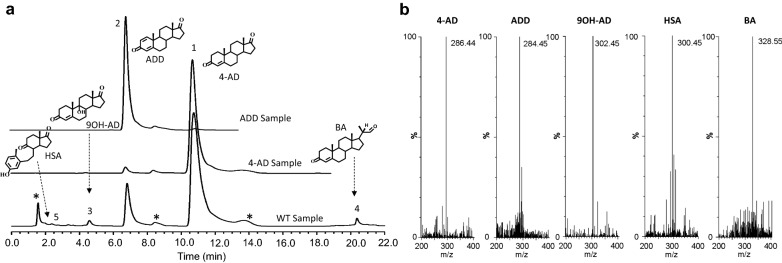
Characterization of major catabolites of the extracts from large-scale HGMS2 fermentation using phytosterol as sole carbon source for 144 h. **a** HPLC profiling of the extracts in comparison with those of standard 4-AD and ADD samples. (1) 4-AD; (2) ADD; (3) 9OH-AD; (4) BA; asterisk: unknown HGMS2 metabolites. **b** Mass spectra of the five major HPLC peaks

The wild-type HGSM2 strain has been used in the pharmaceutical industry to produce 4-AD with a reasonable conversion rate and yield using phytosterol as a substate in an 80 m^3^ fermenter. A typical fermentation process was carried out for 7–10 days, during which time the process was monitored by TLC and HPLC assays until the substrates were completely converted. Figure 2a shows the HPLC profile of an extract from the HGMS2 fermentation broth after seven days of phytosterol fermentation. As shown in Fig. 2a, multiple products were accumulated during fermentation. Two major products were 4-AD and ADD, which were readily identified by comparison with the HPLC profiles of the standard 4-AD and ADD samples. Other minor peaks were identified as 9OH-AD, HSA and BA. All five products were further confirmed by mass spectrometry (Fig. 2b). Based on HPLC profiling, the contents of the five products were 78.21%, 10.33%, 1.43%, 3.85 and 0.54% for 4-AD, ADD, 9OH-AD BA and HSA, respectively, whereas the remaining contents were unknown metabolites unrelated to phytosterol catabolism.

### Construction of KstD-knockout and Ksh-knockout mutants

**Fig. 3:**
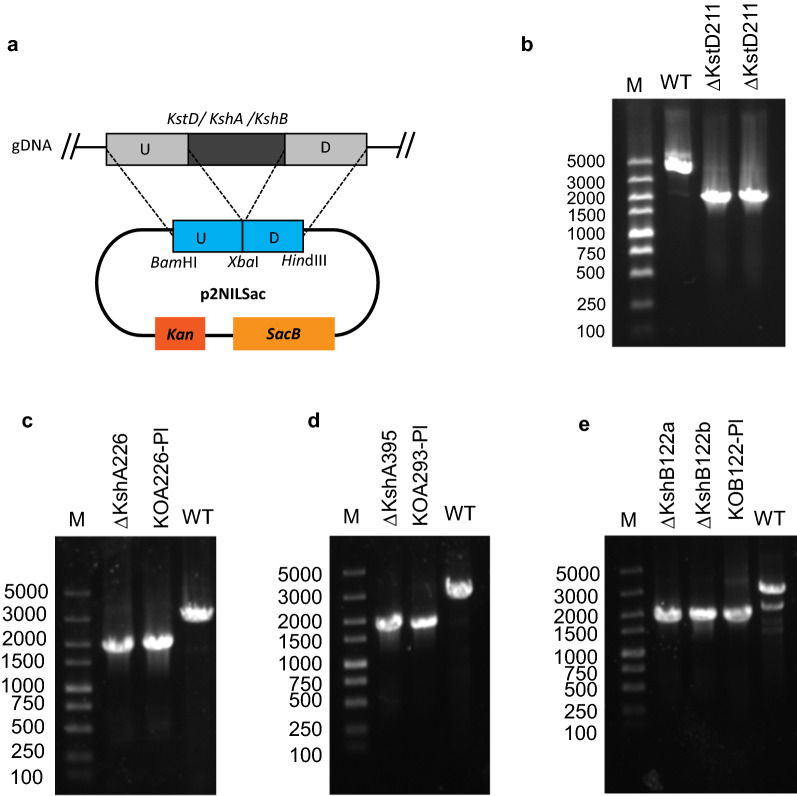
Knockout of the *kstd* and *ksh* genes from the HGMS2 strain. **a** A homologous recombination vector was constructed based on p2NIL plasmid. U: the upstream sequence; D: the downstream sequence. **b** PCR confirmation of putative *kstd*-knockout colonies, in comparison with that for the WT strain. M: DNA marker; WT: PCR products amplified from the HGMS2; ΔKstD211: two putative *kstd*-default mutants. **c–e** PCR confirmation of putative *ksh*-knockout colonies, in comparison with that for the WT strain. M: DNA marker; ΔKshA226: a putative HGMS2^*ΔkstD211 + ΔkshA226*^ mutant; KOA226-Pl: the plasmid p2NIL-Sac-ΔKshA226; WT: the HGMS2 strain; ΔKshA395: a putative HGMS2^*Δkstd211 + ΔkshA395*^ mutant; KOA395-Pl: the plasmid p2NIL-Sac-ΔKshA395; ΔKshB122a and ΔKshB122: two putative HGMS2^*Δkstd211 + ΔkshB122*^ mutants; KOB122-Pl: the plasmid p2NIL-Sac-ΔKshB122

As indicated above, active KstD and Ksh enzymes diminished the accumulation of 4-AD via the formation of 9,10-secosteroid during phytosterol fermentation by the HGMS2 strain. Thus, we attempted to knock out these genes from the genome of the HGMS2 strain by homologous recombination approaches. To construct a homologous recombination vector with an appropriate antibiotic marker, we examined whether the HGMS2 strain had the ability to resist common antibiotics that can be deactivated by bacterial proteins. Although many mycobacterial species are resistant to a variety of antibiotics [59, 60], as shown in Additional file 1: Table S4, we found that the HGMS2 strain was not resistant to any tested antibiotics including kanamycin (Kan), tetracycline (Tc), streptomycin (Stm), spectinomycin (Spm), ampicillin (Amp) and zeocin (Zn). Thus, we chose Kan as the first selection marker and the sucrose lethality gene (*SacB*) [61] as the secondary selection marker to construct gene knockout/knockin vectors. Using the p2NIL vector that contains a Kan gene as a template, we inserted a synthetic *SacB* gene into the p2NIL vector behind the *Kan* gene. The resultant vector was named p2NILSacB (Fig. 3a).

To use the pNILSac vector to knock out one kstd211 and three *ksh* genes from the HGMS2 strain, each DNA fragment containing around 1000 bp from both sides of a target gene was amplified with PCR and inserted into the *Bam*H I and *Hin*d III sites in the p2NILSac vector. Four recombinant vectors were constructed to delete the *kstd211*, *kshA226*, *kshA395* and *kshB122* genes from the HGMS2 genome and then confirmed by PCR (Additional file 1: Figure S3) and DNA sequencing. The four vectors were named p2NIL-SacB-ΔKstD211, p2NIL-SacB-ΔKshA226, p2NIL-SacB-ΔKshA395 and p2NILSacB-ΔKshB122 (Table 1).

**Table 1:** Lists of homologous recombinant knockout and knockin vectors

Mutation	Vector	Targeted gene	Starting strain
KO	p2NIL-SacB-ΔKstD211	*kstd211*	WT
KO	p2NIL-Sacb-ΔKshA226	*kshA226*	HGMS2^*Δkstd211*^
KO	p2NIL-SacB-ΔKshA395	*kshA395*	HGMS2^*Δkstd211*^
KO	p2NIL-SacB-ΔKshB122	*kshB122*	HGMS2^*Δkstd211*^
KI	p2NIL-SacB-KstD2	*kstD211*	HGMS2^*Δkstd211*^ ^*ΔkshB122*^
KI	p2NIL-SacB-KshA51	*kshA226*	HGMS2^*Δkstd211*^

Because ADD was a major by-product produced by active KstD211 enzyme during phytosterol fermentation with the wild-type HGMS2 strain, we knocked out the *kstd211* gene from the HGMS2 genome in the first stage and then sequentially knocked out *ksh* genes from the *kstd*-knockout mutant. The knockout of the *kstd211* gene was done by electroporation of the p2NIL-SacB-ΔKstD plasmid into fresh HGMS2 competent cells, followed by a two-step selection with kan- and sucrose-based methods (see “Materials and methods”). Positive recombinant colonies were identified with colony PCR. After the *kstd211* gene was deleted from the HGMS2 genome, the size of the PCR product became much shorter (1600 bp) than that of the wild-type strain (3600 bp). As shown in Fig. 3b, two positive colonies were identified, and DNA sequencing results confirmed that the *kstd211* gene was knocked out from both clones. We arbitrarily selected one of these two colonies for further investigation and named it HGMS2^*Δkstd211*^.

As mentioned above, the HGMS2 strain contains two pairs of KshA/KshB enzyme components sharing one single KshB enzyme. To explore the importance of individual Ksh enzymes in the formation of 9OH-AD, we knocked out each *ksh* gene from the HGMS2^*Δkstd211*^ mutant using the same knockout strategy that was used togenerate the HGMS2^*Δkstd211*^ mutant. As shown in Fig. 3c and e, positive recombinant colonies were identified by colony PCR. DNA sequencing also confirmed that these *ksh* genes were removed from the three colonies. The resultant mutants were named HGMS2^*Δkstd211+ΔkshA226*^, HGMS2^*Δkstd211+ΔkshA395*^ and HGMS2^*Δkstd211+ΔkshB122*^.

### Effect of KstD211 and Ksh enzymes on HGMS2 phytosterol transformation

**Fig. 4:**
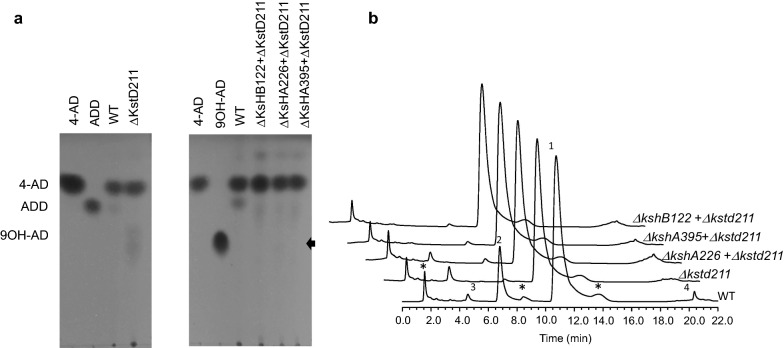
Characterization of *kstd*-knockout and *ksh*-knockout mutants for phytosterol fermentation in small-scale fermentation. **a** TLC assay of the catabolites by *kstd*-knockout and *ksh*-knockout mutants during phytosterol fermentation, compared with those of the WT strain and the standard samples of 4-AD, ADD and 9OH-AD. ΔKstD1, ΔKshB122, ΔKshA226 and ΔKshA395 refer to the HGMS2^*Δkstd211*^, HGMS2^*Δkstd211 + ΔkshB122*^, HGMS2^*Δkstd211 + ΔkshA226*^ and HGMS2^*Δkstd211 + ΔkshA395*^ mutants, respectively. Weak 9OH-AD spots for the samples from the HGMS2^*Δkstd211*^, and HGMS2^*Δkstd211 + ΔkshA226*^ mutants are marked by arrows. **b** HPLC profile of the extracts from the phytosterol fermentation by three double mutants for 144 h compared with those of the WT strain and the HGMS2^*Δkstd211*^ mutant. (1) 4-AD; (2) ADD; (3) 9OH-AD and (4) BA. Asterisk: unknown HGMS2 metabolites when the strain was cultured without phytosterol

To evaluate the effects of *kstd211* and *ksh* knockouts on phytosterol transformation in the HGMS2 strain, small-scale phytosterol fermentation was carried out for the HGMS2 strain and its knockout mutants. First, we tested the HGMS2^*Δkstd211*^ mutant. After phytosterol fermentation for 7 days, the fermentation broth was extracted with a solvent and evaluated byTLC assay. As shown in Fig. 4a, the HGMS2^*Δkstd211*^ mutant abolished ADD production compared with that of the WT strain. However, 9OH-AD was detected on the TLC plate. Notably, knockout of the *kstd211* gene slightly increased the conversion of 4-AD to 9OH-AD (Fig. 4a). Furthermore, the extract was diluted to the same concentration as that of the WT sample based on the TLC density and subjected to HPLC assay. As shown in Fig. 4b, knockout of the *kstd211* gene caused the HGMS2^*Δkstd211*^ strain to be almost free of ADD (peak 2 in Fig. 4b), and we once again observed that the yield of 9OH-AD increased (peak 3 in Fig. 4b). It was reasonable that the accumulation of 9OH-AD occurred when the KstD211-involved formation of 9,10-secosteroid was blocked.

We further tested the effects of the three *ksh* knockout mutants on phytosterol transformation on a small scale. As shown in Fig. 4a, knockout of the three *ksh* genes from the HGMS2 strain exerted a distinct effect on the accumulation of 9OH-AD. A weak spot was found for the sample from the HGMS2^*Δkstd211+ΔkshA226*^ mutant, while no detecTable 9OH-AD spots were visualized for the other two mutants. This observation was consistent with our previous finding that KshA226 was inactive for 4-AD production, in contrast to KshA395 and KshB122 [55]. As shown in Fig. 4b, HPLC profiling of these extracts clearly indicated that the production of 9OH-AD was completely blocked during phytosterol transformation in the HGMS2^*Δkstd211+ΔkshA395*^ and HGMS2^*Δkstd211+ΔkshB122*^ mutants. Notably, the production of ADD was also blocked in these two mutant strains. No detecTable 9OH-AD was observed at the end of 7 days of fermentation and 4-AD significantly accumulated in the HGMS2^*Δkstd1+ΔkshB122*^ mutant with a conversion rate of 47.2% and a yield of 5.62 g/L when starting from 10 g/L of phytosterol (Table 2). Because the deletion of *kshB122* could simultaneously disable the functions of the Ksh226 and KshA395 enzymes, we selected the HGMS2^*Δkstd1+ΔkshB122*^ mutant for pilot scale fermentation.

**Table 2:** Comparison of conversion rates and yields of 4-AD, ADD and 9OH-AD from phytosterol by a variety of *Mycobacterium* strains

Strain	Phytosterol concentration (g/L)	Yield			Conversion rate	References
		4-AD (g/L)	ADD (g/L)	9OH-AD (g/L)		
4-AD-producing strains						
* Mycobacterium* sp. ATCC 25795	15	6.85	5.57	N/A	45.67% (4-AD); 37.13% (ADD)	[64]
* Mycobacterium* sp. B-3683	5	2.35	N/A	N/A	47%	[65]
* Mycobacterium* sp. B-3805	20	7.4	N/A	N/A	37%	[51]
* Mycobacterium smegmatis* MC^2^ 155	10	8.8-9.0	1.0-1.1	N/A	N/A	[26]
* Mycobacterium* sp. HGMS2	10	5.62	1.30	0.30	47.2%	This work
* Mycobacterium* sp. HGMS2	80	31.4	6.26	0.88	40.6%	This work
* Mycobacterium* sp. HGMS2^*Δkstd211 + ΔkshB122*^	10	6.22	NO	NO	51.6%	This work
* Mycobacterium* sp. HGMS2^*Δkstd211 + ΔkshB122*^	80	38.3	NO	NO	48.7%	This work
ADD-producing strains						
* Mycobacterium neoaurum* NwIB-04	15	0.096	4.94	N/A	32.93%	[32]
* Mycobacterium neoaurum* NwIB-01	0.4	N/A	0.174	N/A	43.5%	[31]
* Mycobacterium sp.* HGMS2^*kstd2 + Δkstd211+ΔkshB122*^	10	NO	4.12	NO	44.2%	This work
* Mycobacterium sp.* HGMS2^*kstd2 + Δkstd211+ΔkshB122*^	80	NO	34.2	NO	42.5%	This work
9OH-AD-producing strains						
* Mycobacterium sp.* MS136	13	N/A	N/A	6.0	40%	[21]
* Mycobacterium neoaurum* 25795 mutant	20	N/A	N/A	10.27	51.2%	[63]
* Mycobacterium neoaurum ATCC 25795* mutant	15	N/A	N/A	6.20	41.33%	[30]
* Mycobacterium sp.* HGMS2^*kshA51 + Δkstd211+ΔkshA226*^	10	NO	NO	4.2	44.2%	This work
* Mycobacterium sp.* HGMS2^*kshA51 + Δkstd211+ΔkshA226*^	80	NO	NO	37.2	40.3%	This work

*N/A* data not available, *SD* significant detectable, *NO* not observable

### Pilot-scale phytosterol fermentation with the *HGMS2*^*Δkstd211+ΔkshB122*^ mutant

Pilot-scale phytosterol fermentations using both the wild-type HGMS2 strain and the HGMS2^*Δkstd211+ΔkshB122*^ mutant were conducted in a 15 L fermenter supplied with 10 L fermentation medium (see “Materials and methods”).

**Fig. 5:**
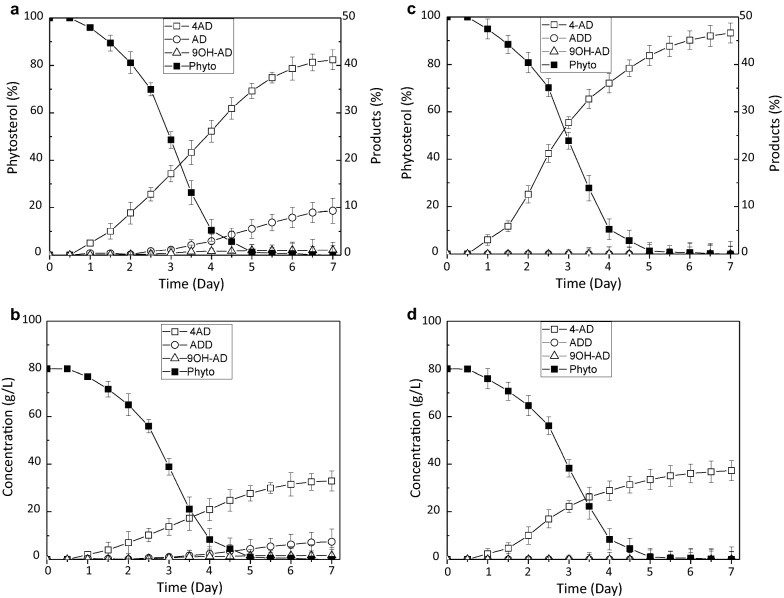
Time course of phytosterol transformation to 4-AD, ADD and 9OH-AD in pilot-scale fermentation. **a**, **b** The conversion rate and yield of phytosterol by the HGMS2 strain, compared with the wild-type strain.; **c**, **b** The conversion rate and yield of phytosterol by the HGMS2^Δ^^*KstD211 + ΔkshB122*^ mutant

As shown in Fig. 5a, the rate of phytosterol conversion to 4-AD increased dramatically within the first 5 days and reached 40.6% after 7 days of fermentation with 80 g/L of phytosterol, while the phytosterol content in the medium decreased. On average, the final yield of 4-AD was 31.4 ± 4.3 g/L (Fig. 5b; Table 2). Accompanying the production of 4-AD was the accumulation of ADD and 9OH-AD also observed With yields of 6.26 g/L and 0.88 g/L on average (Table 2), respectively, after 7 days of fermentation (Fig. 5b), Resulting in total contents of 9.8 and 1.8% on average, respectively. (Fig. 5a).

As expected, the HGMS2^*Δkstd211 + ΔkshB122*^ mutant exhibited an enhanced conversion rate of phytosterol to 4-AD. As shown in Fig. 5c, the rate of phytosterol conversion to 4-AD increased to 48.7% after 7days of fermentation. The final 4-AD yield in the fermentation broth was estimated to be 38.3 ± 4.7 g/L on average (Fig. 5d; Table 2), after transformation from 80 g/L of phytosterol. Notably, ADD and 9OH-AD almost completely disappeared. When compared with the wild-type HGMS2 strain, the mutant strain enhanced the conversion rate and 4-AD yield by 1.2-fold and 1.2-fold, respectively. Moreover, the HGMS2^*Δkstd211 + ΔkshB122*^ mutant completely catabolized the substrate with the same efficiency as the wild-type strain after 7 days of fermentation (Fig. 5).

### Construction of ADD-producing and 9OH-AD-producing mutants

**Fig. 6:**
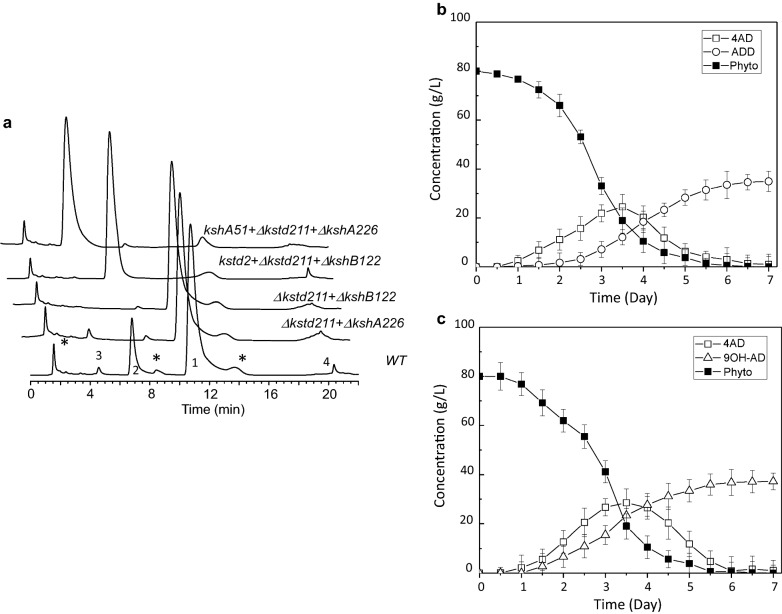
Characterization of ADD- and 9OH-AD-producing mutants during phytosterol fermentation. **a** HPLC profile of the extracts from the phytosterol fermentation by ADD- and 9OH-AD-producing mutants for 144 h compared with those of HGMS2^*Δkstd211 + ΔkshA226*^ and the HGMS2^*Δkstd211 + ΔkshB122*^ mutants. (1) 4-AD; (2) ADD; (3) 9OH-AD and (4) BA. *: unknown HGMS2 metabolites when the strain was cultured without phytosterol. **b**, **c** Time course of ADD and 9OH-AD yields by the HGMS2^*kstd2 + Δkstd1+ΔkshA226*^ and the HGMS2^*kshA51 + Δkstd211+ΔkshB122*^ mutants, respectively

Encouraged by the performance of the HGMS2^*Δkstd211 + ΔkshB122*^ mutant, we attempted to use *kstd*- and *ksh*-default mutants as cell models to construct an ADD-producing strain and a 9OH-AD-producing strain. The KstD2 from *Mycobacterium neoaurum* DSM 1381 is homologous to KstD211 and other KstD enzymes (Additional file 1: Figure S4). The activity of the KstD2 enzyme is much higher than that of the original KstD enzyme in the HGMS2 strain (i.e., KstD211 that converts 4-AD to ADD;Table 3). Therefore, we substituted the *kstd211* gene in HGMS2^*Δkstd1 + ΔkshB122*^ with the *kstd2* gene to generate an ADD-producing mutant strain. This knockin mutant, which was confirmed by PCR (Additional file 1: Figure S5a) and DNA sequencing, was named HGMS2^*kstd2 + Δkstd211+ΔkshB122*^ (Table 3). During tested in a small-scale fermentation system using phytosterol as substrate, the HGMS2^*kstd2 + Δkstd211+ΔkshB122*^ mutant efficiently accumulated ADD within 7 days with a conversion rate of 44.2% and a yield of 4.12 g/L starting from 10 g/L of phytosterol (Table 2). Upon HPLC analysis (Fig. 6a), no detecTable 4-AD and 9OH-AD peaks were observed and all 4-AD generated in the HGMS2^*Δkstd211 + ΔkshB122*^ mutant was eventually transformed to ADD in the HGMS2^*kstd2 + Δkstd211+ΔkshB122*^ mutant. During pilot-scale fermentation, the conversion rate of phytosterol to ADD was 42.5% on average after 7 days of fermentation with 80 g/L of phytosterol. The final ADD yield in the fermentation broth was estimated to be 34.2 ± 5.3 g/L on average (Fig. 6b; Table 2). As shown in Fig. 6b, this ADD-producing mutant completely catabolized the substrate after 5.5 days during pilot-scale fermentation. In this process, 4-AD was an intermediate, and its accumulation started to increase within the first 3.5 days, after which it decreased until complete transformation to ADD had occurred.

**Table 3:** Construction of ADD- and 9OH-AD-producing mutants through knocking in highly-active *kstd* and *ksh* genes, respectively

Product	Starting strain	Original gene		Mutant strain	Knockin gene	
		Enzyme	In vitro Activity (units/mg)		Enzyme	In vitro activity (units/mg)
ADD	HGMS2^*Δkstkd211 + ΔkshB122*^	KstD211	125.7 ± 23.4	HGMS2^*Δkstd211 + ΔkshB122+kstD2*^	KstD2	9238.7 ± 68
9OH-AD	HGMS2^*Δkstd211 + Δksh226*^	KshA395	56.4 ± 7.9	HGMS2^*Δkstd211 + ΔkshA226+kshA51*^	KshA51	532 ± 42

KshA51 is a chimeric KshA enzyme that is generated by KshA1 and KshA5 from *R. rhodochrous* DSM 43269 [57] and is homologous to KshA395 and other KshA enzymes (Additional file 1: Figure S6). KshA51 is much more active than KshA395 for 4-AD (Table 3). To construct a 9OH-AD-producing strain, we replaced the latent *kshA226* gene in the HGMS2^*Δkstd211 + ΔkshA226*^ mutant with a synthetic KshA51 gene that was knocked into the locus of *kshA226* in the HGMS2^*Δkstd211 + ΔkshA226*^ mutant. The resultant mutant was confirmed by PCR (Additional file 1: Figure S5b) and DNA sequencing and then named HGMS2^*kshA51 + Δkstd211+ΔkshA226*^ (Table 3). We examined the HGMS2^*kshA51 + Δkstd211+ΔkshA226*^ mutant for phytosterol transformation during small-scale fermentation for 7 days. The catabolites from the fermentation broth were extracted and evaluated by HPLC. As shown in Fig. 6a, no detecTable 4-AD or ADD was observed at the end of 7 days of fermentation, and 9OH-AD significantly accumulated in this mutant with a conversion rate of 44.2% and a yield of 4.2 g/L when starting from 10 g/L of phytosterol (Table 2). During a pilot-scale fermentation the conversion rate of phytosterol to 9OH-AD was 40.3% on average after 7 days of fermentation (Fig. 6c). As shown in Fig. 6c; Table 2, this 9OH-AD-producing mutant completely catabolized the phytosterol substrate (80 g/L) after 6 days, with an estimated final 9OH-AD yield in the fermentation broth of 37.2 ± 5.3 g/L on average. During phytosterol transformation, 4-AD was an intermediate and its 4-AD accumulation started to increase within the first 3 days, after which it decreased until early completely transformation to 9OH-AD had occurred.

## Discussion

Previously, many *Mycobacteria* and their *kstd*-knockout and/or *ksh*-knockout mutants were investigated for the accumulation of 4-AD, ADD and/or 9OH-AD. Many of these strains exhibited reasonable rates for the transformation of phytosterol with the potential for industrial application (Table 2). However, two major issues remained. Specifically, these *Mycobacterium* strains either continued to produce multiple products that hampered product purification or were investigated at lower concentrations of phytosterol. This indicated that it was likely that the template strains used in these pioneer works contained residual *kstd* and *ksh* genes. Conversely, *Mycobacterium neoaurum* HGMS2 contained fewer *kstd* and *ksh* genes than other bacterial strains [26, 31, 43, 52, 62]. In this study, we first generated a few *M. neoaurum* HGMS2 mutants by knocking out the *kstd* and *ksh* genes. When compared with the wild-type strain, the mutants exhibited improved performance in the conversion of phytosterol to 4-AD. After the *kstd211* gene was knocked out from the HGMS2 strain, the mutant HGMS2^*Δkstd211*^ strain lost its ability to convert 4-AD to ADD; thus, ADD disappeared from the phytosterol fermentation broth. Furthermore, three *ksh* genes were individually knocked out from the HGMS2^*Δkstd211*^ mutant. The three double mutants that were exhibited different abilities to convert 4-AD to 9OH-AD. Specifically, the HGMS2^*Δkstd211 + ΔkshA395*^ and HGMS2^*Δkstd211 + ΔkshB122*^ mutants completely blocked the occurrence of 9OH-AD, while the HGMS2^*Δkstd211 + ΔkshA226*^ mutant still converted 4-AD to 9OH-AD. Therefore, knocking out the *kstd* gene first and then the *ksh* genes benefited the accumulation of 4-AD during phytosterol conversion. Tested in pilot-scale fermentation with a high concentration of phytosterol revealed that the HGMS2^*Δkstd211 + ΔkshB122*^ mutant increased the conversion rate by 1.2-fold to 48.7% on average. resulting in the 4-AD yield transformed by this double mutant increasing to 38.3 ± 8.7 g/L. In our pilot-scale fermentation, the conversion rate and 4-AD yield of our new 4-AD-producing strain only reached 75% of their theoretical values, i.e., 65% and 52.0 g/L, respectively. As shown in Figs. 2a and 6a, it was likely that the HGMS2 and its mutants still transformed phytosterol to BA and other intermediates. Further gene engineering should improve the 4-AD production yield. In our pilot-scale study, we also observed that the viscosity of the fermentation broth increased significantly as the starting concentration of phytosterol increased from 10 to 80 g/L. Nevertheless, we did not observe any significant inhibitory growth by substrate or product (*data not shown*). Thus, we expect that it should be possible to increase the starting concentration of phytosterol in aqueous fermentation medium, when exploring new formulae for fermentation media.

Furthermore, we constructed ADD- and 9OH-AD-producing strains by knocking the genes of active heterogenous KstD and KshA enzymes into the *M. neoaurum* HGMS2 mutants, respectively. The resultant mutant HGMS2^*kstd2 + Δkstd211+ΔkshB122*^ could efficiently and completely convert phytosterol to ADD with an average conversion rate of 42.5% and an average yield of 34.2 ± 5.3 g/L from 80 g/L of phytosterol. The HGMS2^*kshA51 + Δkstd211+ΔkshA226*^ mutants also efficiently converted phytosterol into 9OH-AD with an average conversion rate of 40.3% and an average yield of 37.2 ± 5.2 g/L from 80 g/L of phytosterol. These results were comparable to those obtained with other mycobacterial strains [21, 30, 31, 63]. One of the significant advantages of our mutants was that they generated less impurities. Because these two mutants were made of *kstd-* and *ksh*-knockout strains, further knock outs of other impurity-related genes should improve the ADD and 9OH-AD production yield.

In conclusion, our work provides efficient mutants for the production of 4-AD, ADD and 9OH-AD for industrial application. The gene implementation of *M. *sp. HGMS2 in this work has demonstrated that the HGMS2 strain is an excellent model for engineering microbial cell factories to produce important steroidal compounds. The production of another important pharmaceutical compound, BA, via similar gene engineering methods is currently under investigation in our laboratory.

## Supplementary Information

**Additional file 1.** Supplementary informations include homologous recombinant sequences for knocking out targeted genes in the HGMS2 mutants, primers used for the construction of knockout and knockin vectors, antibiotic resistances of the HGMS2 strain, DNA sequence of KstD2 gene, DNA sequence of KshA51 gene, amino acid alignment of KstD211 with other KstD enzymes, amino acid alignment of KshA51 with the Ksh enzymes from the HGMS2 strain, and experimental data for PCR screening of KO and KI mutants.

## Data Availability

Not applicable.

## Abbreviations

4-AD: 4-Androstene-3,17-dione
9OH-AD: 9α-Hydroxyl-4-androstene-3,17-dione
ADD: 1,4-Androstene-3,17-dione
BA: 21-Hydroxy-20-methylpregn-4-en-3-one
DCPIP: 2,6-Dichlorophenolindophenol
HPLC: High-performance liquid chromatography
HSA: 3-Hydroxy-9,10-secoandrost-1,3,5(10)-triene-9,17-dione
KstD: 3-Ketosteroid-1,2-dehydrogenase
Ksh: 3-Ketosteroid-9α-hydroxylase
TLC: Thin layer chromatography
