# Supplementary material for: Efficient conversion of phytosterols into 4-androstene-3,17-dione and its C1,2-dehydrogenized and 9α-hydroxylated derivatives by engineered Mycobacteria
Source: Microb Cell Fact. 2021 Aug 16;20:158. doi: 10.1186/s12934-021-01653-9 (PMC8365914; doi:10.1186/s12934-021-01653-9)
Supplement: Supplementary file 1 — Additional file 1. Supplementary informations include homologous recombinant sequences for knocking out targeted genes in the HGMS2 mutants, primers used for the construction of knockout and knockin vectors, antibiotic resistances of the HGMS2 strain, DNA sequence of KstD2 gene, DNA sequence of KshA51 gene, amino acid alignment of KstD211 with other KstD enzymes, amino acid alignment of KshA51 with the Ksh enzymes from the HGMS2 strain, and experimental data for PCR screening of KO and KI mutants. [file 12934_2021_1653_MOESM1_ESM.pdf]

Additional information for

Efficient conversion of phytosterols into 4-androstene-3,17-dione and its C1,2-dehydrogenized and 9α-hydroxylated derivatives with engineered *Mycobacteria*

Xin Li[1,#], Tian Chen[1,#], Fei Peng[1,#], Shikui Song[1], Jingpeng Yu[1], Douanla Njimeli Sidoine[1], Xiyao Cheng[1], Yongqi Huang[1], Yijun He[2,*] and Zhengding Su[1,*]

[1]*Key Laboratory of Industrial Fermentation (Ministry of Education), National "111" Center for Cellular Regulation and Molecular Pharmaceutics and Hubei Key Laboratory of Industrial Microbiology, Hubei University of Technology, Wuhan, 430068, China;*

[2]*Hubei Goto Biotech Inc. No. 1 Baiguoshu Road, Shuidu Industrial Park, Danjiangkou, Hubei, 442700, China*

# These authors contribute to this work equally.

[*]**To whom correspondence should be addressed:** Zhengding Su, Email: zhengdingsu@hbut.edu.cn, Tel.: 86-156-23901978, ORCID iD: 0000-0003-3558-001X; Yijun He, Email: heyijun@gotochem.com, Tel.:86-0710-3423123.

Keywords: 1,4-androstadiene-3,17-dione (ADD); 21-hydroxy-20-methylpregn-4-en-3-one (BA); 3-ketosteroid-1,2-dehydrogenase (KstD); 3-ketosteroid-9α-hydroxylase (Ksh); 4-androstene-3,17-dione (4-AD); 9α-hydroxyl-4-androstene-3,17-dione (9OH-AD); *Mycobacterium sp*. strain.

Table S1. Homologous recombinant sequences for knocking out targeted genes in the HGMS2 mutants

Targeted gene	Mutant strain	Experimental data	GenBank Sequence ID & locus
KstD211	*M. sp.* HGMS2$^{\Delta KstD}$	KstD_F_2019-06-11_F11.ab1<br>KstD_F_2019-06-11_F11.seq	GenBank: CP031414.1<br>224137-222716
		KstD_R_2019-06-11_E12.ab1<br>KstD_R_2019-06-11_E12.seq	GenBank: CP031414.1<br>221149-220094
KshA226	*M. sp.* HGMS2$^{\Delta KstD+KshA226}$	226_F_2019-06-11_A02.ab1<br>226_F_2019-06-11_A02.seq	GenBank: CP031414.1<br>237256-236205
		226_R_2019-06-11_A12.ab1<br>226_R_2019-06-11_A12.seq	GenBank: CP031414.1<br>239329-238439
KshA395	*M. sp.* HGMS2$^{\Delta KstD+\Delta KshA395}$	395_F_2019-06-11_B02.ab1<br>395_F_2019-06-11_B02.seq	GenBank: CP031414.1<br>3612419-3611411
		395_R_2019-06-11_E02.ab1<br>395_R_2019-06-11_E02.seq	GenBank: CP031414.1<br>3610261-3609267
KshB122	*M. sp.* HGMS2$^{\Delta KstD+\Delta KshB122}$	122_F_2019-06-04_G06.ab1<br>122_F_2019-06-04_G06.seq	CP031414.1<br>117397-116375
		122_R_2019-06-04_H06.ab1<br>122_R_2019-06-04_H06.seq	GenBank: CP031414.1<br>115318-114331

Table S2. Primers used for the construction of knockout vectors

Target genes	Primers	Sequence	Note
KstD211	Kstd-D-F	CCACAACTCGAGATGACGTTCGGCTACCTCGC	*Xho*I
	Kstd-D-R	ACGAGCAAGCTTCCGACAGTCTCCCACTCGG	*Hin*dIII
	Kstd-U-F	GCAGTAGGATCCCCACAATCTCGGCATACC	*Bam*HI
	Kstd-U-R	CCACAACTCGAGGGTGAGGGCGGCGACCATG	*Xho*I
KshB122	KshB122-D-F	GCTCTAGATGCTCGGGCTAACGTGCAGAC	*Xba*I
	KshB122-D-R	CCCAAGCTTTGACCGGGAGCTCGACGGCG	*Hin*dIII
	KshB122-U-F	CGGGATCCTCGGCGATCCGCACCTTGG	*Bam*HI
	KshB122-U-R	GCTCTAGAAGGCCTGGCCTCATTTCCCTC	XbaI
KshA226	KshA226-D-F	GCTCTAGATGATGACCACGCATGACG	*Xba*I
	KshA226-D-R	CCCAAGCTTCCTGCAGTTGATCGGCCTC	*Hin*dIII
	KshA226-U-F	CGGGATCCGGAAGTGGACAAGGCGACCG	*Bam*HI
	KshA226-U-R	GCTCTAGAGGTGGGCCTCCCGTATCTGG	*Xba*I
KshA395	KshA395-D-F	CCCTCGAGGCGAACCGGCCAACGGCC	*Xho*I
	KshA395-D-R	CGGGATCCGGTCCTTGATGGCGGTCGAGACC	*Bam*HI
	KshA395-U-F	CCAAGCTTCGCTGTTCCACGGCAACGCGA	*Hin*dIII
	KshA395-U-R	CCCTCGAGCTGATCTGCCTAGCACGTTCACGT	*Xho*I

Table S3. Primers used for the construction of knockin vectors

KI gene	Primers	Sequence	Cloning site	Locus
KstD2	KstD2-F	GCTCTAGAATGACTGAACAGGACTACAG	*XbaI*	*KstD211*
	KstD2-R	GCTCTAGATCAGGCCTTTCCAGCGAG	*XbaI*	
KshA51	KshA51-F	GCTCTAGAATGTCCATTGATACC	*Xba*I	*KshA226*
	KshA51-R	GCTCTAGATTAATTTCGATTATGCGG	*XbaI*	

Table S4. Antibiotic resistances of the HGMS2 strain

**Antibiotics**	**Concentration** (μg/ml)	**Negative (-) or Positive (+)**
Kanamycin (Kan)	50	-
Tetracycline (Tc)	10	-
Streptomycin (Spm)	100	-
Spectinomycin (Stm)	50	-
Ampicillin (Amp)	100	-
Zeocin (Zn)	400	-

ATGACCGATCAGAACAACATCACCGTCGACCTCGTCGTCGGCTCGGGTACCGGGATGG
CGGCAGCATTGGCTGCCCACGAGCTGGGAATGTCGACGCTGATCGTCGAGAAGAGCGCCT
ATGTCGGTGGTTCGACGGCTCGCTCCGGCGGTGCCTTCTGGCTTCCCGGCAGCTCCATTCTC
AAGGACGCCGGTTCGGCGGACACTCCGGCCAAGGCGCGCACCTACCTTGAAGCACTCGTC
GGTGACGACGTCTCACCCGAACGCGCACGCACTTTCATCGATCAGATCCCCGCGACCATCG
ACATGTTGCGTCGCACCACCCCGATGAAGTTCATGTGGGCCAAGGGATATTCGGACTACCA
CCCGGAGAGGCCAGGAGGCAGTGCGGTGGGCCGGACCTGTGAGTGTCGCCCGTTCGACAC
TGCGGTCCTCGGTCCAGAGCTGGCGCGGCTACGACCTGGAGTGATGAAGTCATCGTTCCCG
ATGCCGGTCACCGGCGCCGATTACCGTTGGCTGAACCTGATGGCCCGCACCCCGCGCAAGT
CCTGGCCGCGGATCATGCTGCGGGCCATGCAGGGTGTCGGCGGTTTGGCCCTGCGGCGCCG
GTACGCCGCAGGCGGCCAGGCCTTGGCGGCCGGGATGTTCGCCGGCGTGCTGCAGGCGGG
GATCCCGGTGTGGACCGATTCGACGGTGACCGAGCTCATCACCGATGGTGGGCGGGTGACC
GGCGCGCGGGTGCTGCGCGAGGGATCGGCCGTGACCGTCACCGCACGCCGTGGCATCGTG
CTGGCCACCGGCGGTTTCGACCACGAGATGAATTGGCGGCGGAAGTTCCAGTCCGAGCTC
CTCGGTGAACATCTCAGCCTTGGGGCCGAGAGCAATACCGGCGATGGCATCCGGCTCGCCC
AGGACCTGGGCGCAGGCACCGGACTGATGGACCAGGCATGGTGGTTTCCGGCCTTTGCTCC
GCTGCCTGGCGGGGATCCCACCGTGATGCTGGCCGAGCGGTCGCTGCCCGGCTGCCTGCTG
GTAGACCAGACCGGTGAGCGCTTCATCAACGAGGCCACCGACTACATGTCCTTCGGACAGC
AGCTGCTGCGTCGCGAACACGCGGGCAATCCGGTCGAGACGATGTGGATGATCTTCGATCA
GCGCTACCGGAACAGCTATCTGCTTGCCGCCGAACTATTTCCACGAATGCCGATCCCACAG
AGTTGGTACGACGCCGGGATCGCGCACCGCGGCACGGATGCGGAAGCACTGGGCCGCCAG
ATCGGTTTCGATCCCGCGACGTTGGTCGCCACGATCGAGCGGTTCAACGGACTCGCCGATG
CCGGTGTCGACGCCGACTTCCAGCGCGGCGCGAGCGCCTACGACCGCTACTACGGCGACC
CGACGATCACGCCCAACCCGAACCTGCGACCGCTGGATCCCGGCCCGCTGTACGCCGTCAA
GGTCGTGCTGAGCGACCTGGGCACCTGTGGTGGGGTCCTGTGCGACGTGAACGGCCGGGT
TCTGCGCGAAGACGGAGTGCCCATCGACGGTCTGTACGCGATCGGCAATACCGCGGCCAAC
GCATTCGGCAAGACCTACCCGGGCGCGGGCGCGACCATCGCGCAGGGGCTGGTGTACGGC
CATGTTGCCGCGCAGCATGCCGCCGGACACACCTGA

Fig. S1. DNA sequence of *KstD2* gene *from Mycobacterium neoaurum* DSM1381

ATGTCCATTGATACCGCACGCTCTGGTTCGGACGACGATGTCGAGATCCGCGAAATTCAAG
CTGCTGCAGCTCCTACACGCTTCGCCCGCGGGTGGCACTGCTTGGGCTTATTGCGCGACTTT
CAGGATGGCAAACCCCACTCCATCGAGGCGTTTGGGACTAAACTGGTGGTTTTCGCCGATT
CGAAAGGGCAGTTGAACGTTCTGGACGCCTATTGTCGTCACATGGGAGGAGACTTGAGCC
GCGGCGAGGTTAAGGGTGATTCTATTGCTTGTCCCTTTCATGATTGGCGCTGGAATGGAAAA
GGAAAATGTACTGACATTCCCTACGCCCGCCGTGTGCCTCCGATTGCAAAGACACGCGCTT
GGACTACTTTAGAGCGTAACGGTCAACTGTATGTATGGAATGACCCCCAAGGAAATCCTCC
GCCGGAGGATGTGACTATCCCTGAAATTGCTGGGTACGGAACTGATGAGTGGACCGACTGG
AGTTGGAAATCACTTCGCATCAAAGGTTCTCACTGTCGTGAGATTGTCGATAACGTAGTAGA
CATGGCCCATTTTTTTTATATTCACTATTCTTTCCCGCGCTACTTTAAGAACGTTTTTGAAGGC
CACGTTGCTAGCCAATTCATGCGCGGTCAGGCGCGTGAAGATGTTATTTCGGGTACCAACTA
CGATGATCCTAATGCAGAATTACGCAGCGAGGCAACATACTTCGGCCCTAGCTACATGATCG
ATGACTTGGAAAGCGACGCGAATGGACAAACAATTGAGACCATCTTAATTAACTGTCACTA
CCCGGTGTCGAACAACGAGTTCGTTTTGCAGTACGGCGCAATCGTGAAAAAGTTGCCCGG
CGTCAGTGATGAGATCGCGGCCGGGATGGCTGAGCAATTCGCGGAAGGAGTTCAGCTTGG
CTTTGAACAAGACGTGGAGATTTGGAAAAATAAGGCACCGATCGATAATCCCCTGTTGAGC
GAAGAGGACGGGCCTGTCTACCAATTGCGTCGTTGGTACCAACAATTTTACGTAGACGTCG
AAGACATCACTGAAGATATGACAAAGCGCTTCGAGTTCGAGATCGACACTACACGCGCCGT
TGCCAGCTGGCAGAAGGAGGTCGCTGAGAATCTTGCCAAGCAGGCAGAAGGCTCAACCGC
TACTCCCTAAGAATTCGAGCTCGGCGCGCCTGCAGGTCGACAAGCTTGCGGCCGCATAATG
CTTAAGTCGAACAGAAAGTAATCGTATTGTACACGGCCGCATAATCGAAATTAA

Fig. S2. DNA sequence of *R. rhodochrous* DSM43269 *KshA51* gene

a) **b)** **c)**

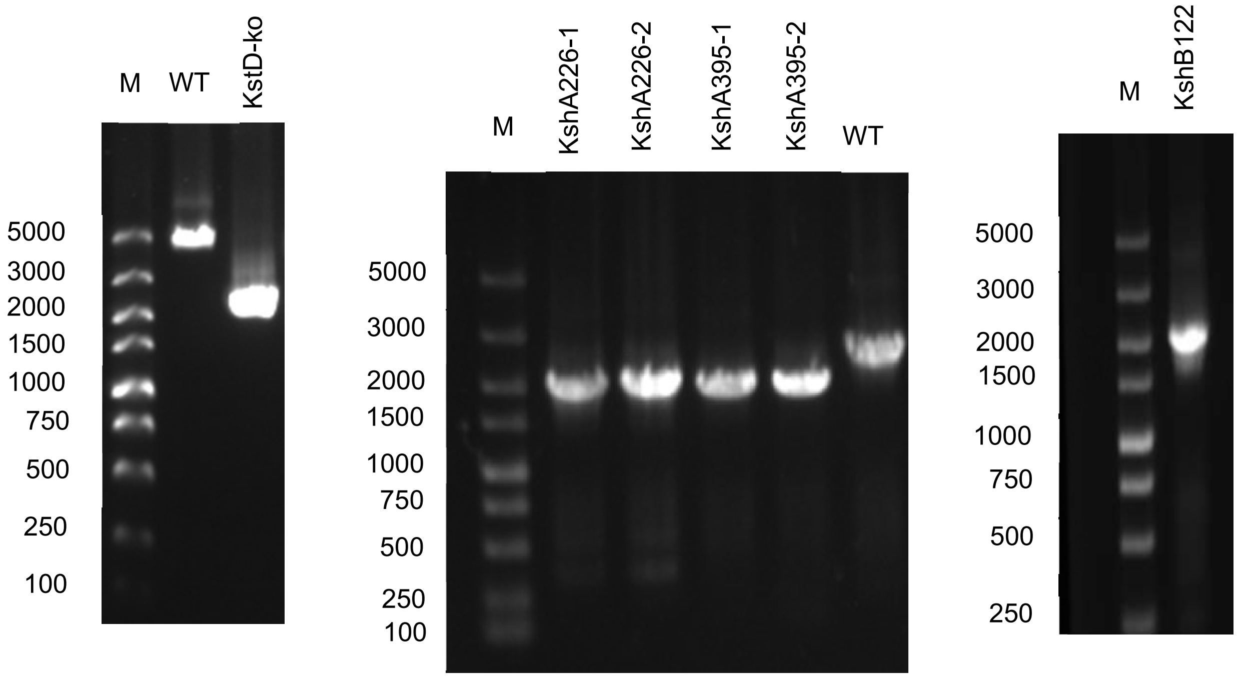

Fig. S3. PCR screening of KO mutants. **a**. PCR verification of the p2NIL-SacB-ΔKstD vector. M: DNA marker; WT: PCR product including KstD gene and its upstream and downstream sequences amplified from the *M. sp.* HGMS2 genome; KstD-ko: PCR product amplified from the p2NIL-SacB-ΔKstD vector. **b**. PCR verification of p2NIL-SacB-ΔKshA226 and p2NIL-SacB-ΔKshA395 vectors. M: DNA marker; KshA226-1 and KshA226-2: two PCR products amplified from putative colonies harboring the p2NIL-SacB-ΔKshA226 vector; and KshA395-1 and KshA395-2: two PCR products amplified from putative colonies harboring the p2NIL-Sac-ΔKshA395 vector. WT: PCR product including the KshA226 gene and its upstream and downstream sequences amplified from the *M. sp.* HGMS2 genome. **c**. PCR verification of the knockout the p2NIL-SacB-ΔKshB122 vector. M: DNA marker and KshB122: PCR product amplified from putative colony harboring the p2NIL-SacB-ΔKshB122 vector.

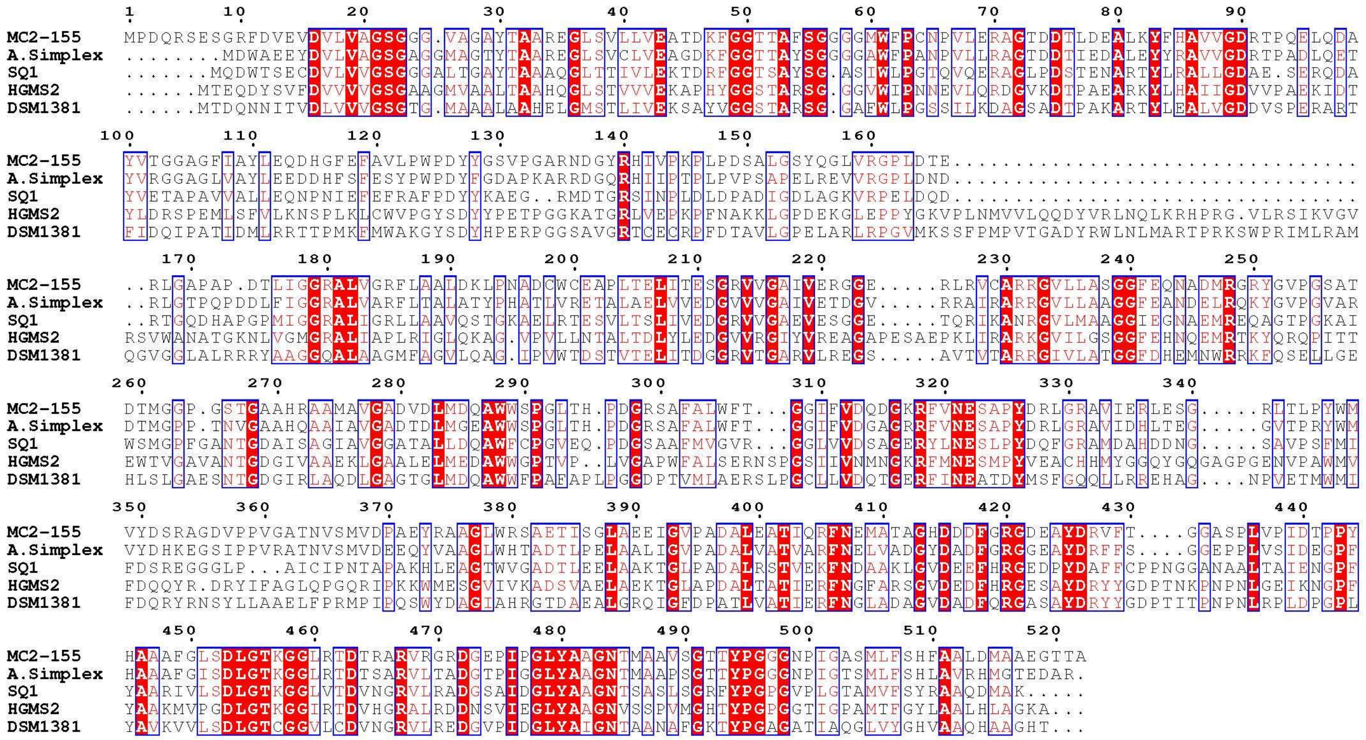

Fig. S4. Amino acid alignment of KstD211 with other KstD enzymes. DSM1381: *Mycobacterium sp.* DSM1381; MC^2 155: *Mycobacterium sp.* MC^2 155; *A. simplex*: *Arthrobacter simplex* and SQ1: *Rhodococcus erythropolis* SQ1. Solid and open frames indicated identical and similar residues, respectively.

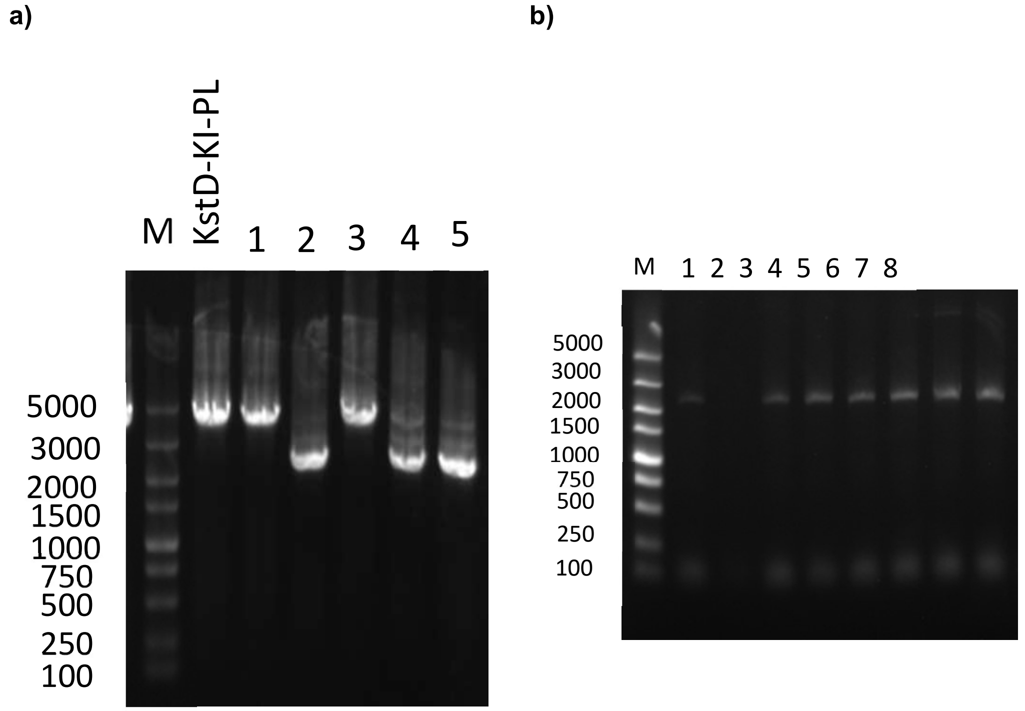

Fig. S5. PCR screening of KI mutants. **a**. KstD KI mutants. M: DNA marker; KstD-KI-PL, KstD2 knockin vector; 1-5, five knockin colonies. **b**. KshA51 KI mutants. M: DNA marker; 1-8, five knockin colonies.

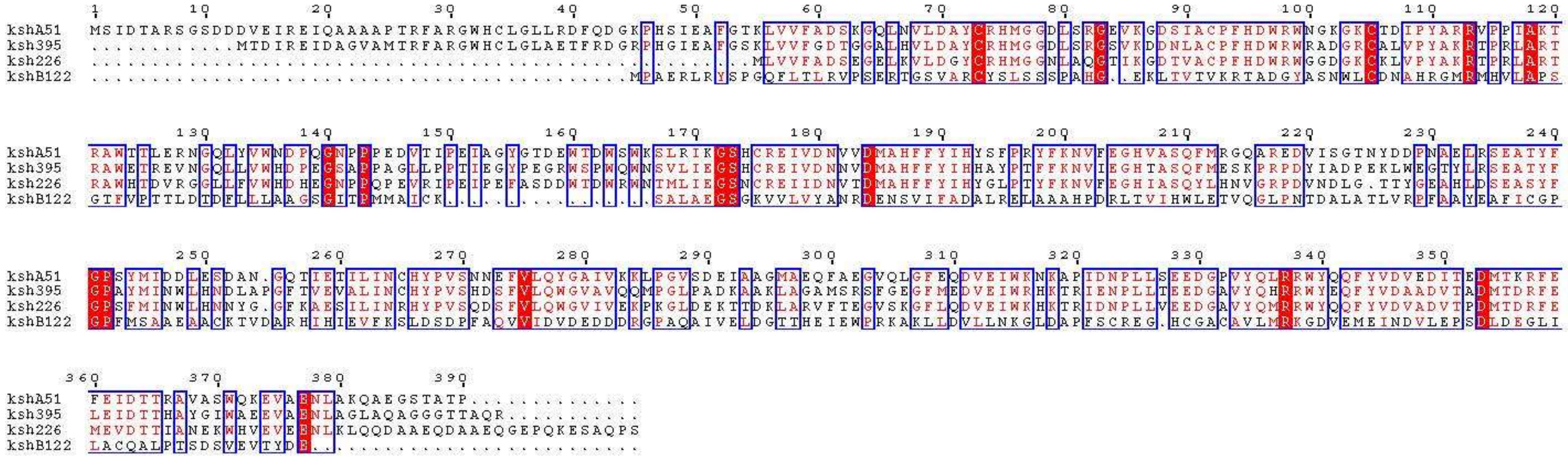

Fig. S6. Amino acid Alignment of KshA51 with the Ksh enzymes from the HGMS2 strain.
